# Drug use in children hospitalized with cardio-rheumatologic diseases in Andijan, Uzbekistan: a cross-sectional descriptive study

**DOI:** 10.1186/s40360-016-0051-3

**Published:** 2016-02-25

**Authors:** Umida Ganieva, Ibrohim Alimdjanov, Marifat Ganieva, Tuhtasin Abdunazarov

**Affiliations:** Master’s Degree Student in Cardiology and Rheumatology, Andijan State Medical Institute, Andijan, Uzbekistan; Department of Hospital and Policlinic Pediatrics, Andijan State Medical Institute, Andijan, Uzbekistan; Department of Clinical Pharmacology, Andijan State Medical Institute, Andijan, Uzbekistan

**Keywords:** Cardiovascular drugs, Cardio-rheumatologic diseases, Congenital heart diseases, Drug use, Pediatrics

## Abstract

**Background:**

No studies have been conducted on rational drug use among children in Uzbekistan. This study aimed to analyze drug uses based on pharmaco-epidemiologic (PE) data from Regional Children’s Multi-Profile Medical Centre (RCMPMC) in Andijan, Uzbekistan. Our study assessed drug usage in children with cardiovascular (CV) diseases, without intervening in the treatment processes or in the course of the diseases.

**Methods:**

Subjects were 853 children aged 0 to 180 months (median age, 60 months; inter-quartile range, 24–108 months) who were hospitalized in the department of Cardiology and Rheumatology in RCMPMC from January to December, 2013 and were prescribed one or more drugs during hospitalization. Drugs used for a different disease or medical condition, given in a different way and/or given in a different dose were analyzed and considered to be irrational drugs.

**Results:**

The most commonly used medications among 10 drug groups prescribed by the doctors of RCMPMC were as follows: anti-arrhythmic (aspartic acid - 54.0 %), glycosides (digoxin - 44.0 %), diuretics (furosemide - 34.0 %), vitamins (ascorbic acid - 25.0 %), steroid anti-inflammatory drugs (prednisolone - 19.0 %), non-steroid anti-inflammatory drugs (diclofenac - 17.0 %), antibiotics (amoxicillin - 16.0 %), non-steroid anabolic drugs (potassium orotas - 14.0 %) and angiotensin-converting enzyme inhibitors (captopril - 11.0 %).

**Conclusions:**

The study found that irrational drug schemes were quite frequent among pediatric CV patients and they are most frequent in children aged 2–3 years and younger.

## Background

Hospitalized pediatric patients with congenital and acquired heart and rheumatic diseases are prone to influence by both the advantages and risks of inpatient care. The risks may be associated with undesirable drug effects [1] caused by either off-label or irrational drug prescription. Such attitude to treatment increases the cost of care, frequency of side effects and the duration of hospitalization [2, 3].

According to the studies from different countries, 0.15 to 93.6 % of patients are treated with various kinds of errors, including prescribing, transcribing and administration [4–6]. Majority of medicines prescribed have not been studied properly and do not have direct indications to be used in pediatric practice. Most treatments are decided only by clinical experience and doctors’ own observations. Evidence from clinical trials is not used. Drug calculation is disproportioned by using adult doses [7, 8]. Early estimates showed that medical practice based on scientific evidence was as low as 10 to 21 % [9]. The reasons for insufficient information from pharmacological clinical trials conducted in pediatric patients are high diversity among few patients resulting in an absence of statistical power, difficulty in obtaining consent from parents, and lack of resources in pediatric research organizations.

Enough studies were performed to investigate the prescribing trends in adults [10, 11], whereas pediatricians lack such detailed information on most of the drugs [12, 13]. Therefore, children are very vulnerable to possible harmful drug effects, and there are not enough studies on drug use in children [2].

Drug use can be evaluated retrospectively by analyzing prescriptions called “Drug Utilization Study or Research (DUR)” which is considered as an essential part of utilization-oriented pharmaco-epidemiology (PE) [14]. PE assesses drug effects in large heterogeneous population of patients over a long period of time without intervening into the treatment process and without affecting the course of the diseases. DUR developed quickly during the following 30 years [14]. Especially rapid progress was noticed in Australia, Africa, Latin America and South and Middle East, and the number of papers which were published in English increased from 70 in 1973 to 486 in 2000 [13, 15, 16]. However, Asian countries, in particular the Central Asia, have no works in PE until now.

The prevalence of congenital heart diseases (CHD) was reported as 8 per 1,000 live births in the world between 1930 and 1990 (0.6 per 1,000 live births −95 % confidence interval (CI): 0.4 to 0.8 in 1930–1934; 9.1 per 1,000 live births −95 % CI: 9.0 to 9.2 after 1995) [17]. Total CHD prevalence was around 9.3 in 2010 [17]. However, Asia has higher prevalence (9.3 per 1,000 live births) in comparison to Europe (8.2 per 1,000 live births) and North America (6.9 per 1,000 live births) [17]. Annually, 1.35 million children are born with CHD [17] and it is one of the biggest global health burdens, as it affects nearly 1 % of live births and reaches $6 billion in acute care cost alone annually [18]. In Uzbekistan, 10 % of all mortality cases are due to congenital anomalies. Although CHD constitute one third of anomalies, complete estimates are unavailable because the data is scarce in Uzbekistan [17, 19]. If no treatment is carried out, 60 % of children with CHD die within the first 2 years. The fact is widely recognized that there are more lethal cases in developing countries [7, 20]. Therefore, it is vital to organize proper treatment both by surgery and by rational medication procedures. We carried out utilization-oriented PE analysis by using DUR to analyze drug use in a pediatric population hospitalized with congenital and acquired heart and rheumatic diseases in the department of Cardiology and Rheumatology in Regional Children’s Multi-Profile Medical Centre (RCMPMC) in Andijan, Uzbekistan. Another aim of our study is to contextualize the need to develop reasonable national estimates of pediatric in-patients treated with a variety of drugs. The lack of such estimations in recent times is hampering the monitoring of hospital drug usage patterns and adverse drug events associated with a certain drug [21].

## Methods

Data for this study were collected from case histories of all patients who were hospitalized in the department of Cardiology and Rheumatology of RCMPMC in Andijan, Uzbekistan from January until December, 2013. The quality and reliability of data were assured by the joint work of Andijan State Medical Institute and the department of Cardiology and Rheumatology of RCMPMC. The hospital provided all necessary information on demographics, diagnoses and all procedures that the patients underwent during the period of hospitalization.

Subjects were children aged 180 months or younger with congenital and acquired heart and rheumatic diseases and received one or more drugs. All drugs prescribed to the study subjects were counted and classified as NSAIDs (non-steroid anti-inflammatory drugs), SAIDs (steroid anti-inflammatory drugs), vitamins-microelements, antibiotics (cephalosporins, penicillins, amino glycosides, macrolides, sulfonamides, lincosamides, fluoroquinolones), antihistamines, bronchodilators, diuretics, antispasmodic, CV drugs (including β-adrenergic blocking agents, glycosides, angiotensin-converting enzyme (ACE) inhibitors) and other drugs such as antivirus, sedative, immune-stimulators and immune-suppressants. We calculated the minimum drug dose per kg for each drug category and estimated whether the drug was used within the approved age range and with indications for pediatrics (rational). For drugs without exact data of dose per kilogram, we considered the age of patients. Demographic and diagnostic variables and the number of total prescribed drugs were summarized using frequencies and percentages for categorical variables and medians and inter-quartile ranges for continuous variables. All analyses were performed using Statistical Package for Social Science (SPSS) version 22 (SPSS Inc. Chicago, IL, USA) and statistical significance was considered at *p* < 0.05.

Approval was obtained from Ethical Committee of Andijan State Medical Institute on June 1, 2014. The Ethical Committee agreed that informed consent was not needed to be taken from patients because no personal data were recorded.

## Results

Table 1 shows drug usage according to selected characteristics in children hospitalized with congenital and acquired heart and rheumatic diseases. Our study population was comprised of patients with diverse diagnostic backgrounds and treatments. Some patients were treated only by medication and the others were treated by surgery followed by medication. Patients with CHD comprised the largest proportion of our study population (frequency, 76.0 %) and only 18.0 % of the patients underwent operations.

**Table 1:** Drug use according to selected characteristics in children hospitalized with congenital and acquired heart diseases (*n* = 853)

Characteristics	Irrational drug use		
	Yes	No	Total
	Number (%)	Number (%)	Number (%)
Gender			
Male	274 (58.2)	197 (41.8)	471 (100)
Female	221 (57.9)	161 (42.1)	382 (100)
Residence			
Urban	88 (54.7)	73 (45.3)	161 (100)
Rural	407 (58.8)	285 (41.2)	692 (100)
Age (years)			
≤ 1	136 (76.8)	41 (23.2)	177 (100)
2–3	110 (67.1)	54 (32.9)	164 (100)
4–6	100 (50.8)	97 (49.2)	197 (100)
7–11	95 (52.5)	86 (47.5)	181 (100)
12–15	54 (40.3)	80 (59.7)	134 (100)
Length of hospitalization (days)			
1–7	205 (50.5)	201 (49.5)	406 (100)
≥ 8	290 (64.9)	157 (35.1)	447 (100)
Diagnosis			
Congenital heart diseases treated by surgery + drug therapy			
Yes	81 (53.6)	70 (46.4)	151 (100)
No	414 (59.0)	288 (41.0)	702 (100)
Congenital heart diseases treated only by drug therapy			
Yes	331 (66.9)	164 (33.1)	495 (100)
No	164 (45.8)	194 (54.2)	358 (100)
Acquired cardiovascular diseases treated by drug therapy			
Yes	398 (67.9)	188 (32.1)	586 (100)
No	97 (36.3)	170 (63.7)	267 (100)
Rheumatic diseases treated by drug therapy			
Yes	88 (45.1)	107 (54.9)	195 (100)
No	407 (61.9)	251 (38.1)	658 (100)
Weight (kg)			
≤ 10	182 (75.5)	59 (24.5)	241 (100)
11–19	166 (56.7)	127 (43.3)	293 (100)
≥ 20	147 (46.1)	172 (53.9)	319 (100)
Number of prescribed drugs			
1–4	65 (21.4)	239 (78.6)	304 (100)
5	115 (56.4)	89 (43.6)	204 (100)
6	153 (86.9)	23 (13.1)	176 (100)
7–14	162 (95.9)	7 (4.1)	169 (100)
All subjects	495 (58.0)	358 (42.0)	853 (100)

CV diagnoses are shown in Table 2. Evaluation of the median age for each group of diagnosis showed that patients with atrioventricular canal were the youngest group (median age, 12 months; inter-quartile range, 5–36 months) and patients with rhythm disorders were the eldest group (median age, 156 months; inter-quartile range, 132–168 months). Juvenile rheumatoid arthritis was the most frequent diagnosis in the group of rheumatic diseases with median age of 108 months (inter-quartile range, 72–144 months). Mesocardia, mitral valve stenosis, scleroderma, non-rheumatic carditis and recurrent rheumatic fever were diagnosed only in 0.1 % of patients hospitalized in the department of Cardiology and Rheumatology.

**Table 2:** Frequency distribution of diagnosis in patients with congenital and acquired heart diseases (*n* = 853)

Diagnosis	Number (%)	Age	
		Median	(IQR)^a^
Congenital heart diseases	646 (75.7)	36	(12–60)
Acquired cardiovascular diseases	586 (68.7)	36	(12–72)
Rheumatic diseases	195 (22.9)	96	(60–144)

^a^Inter-quartile range in month

Table 3 shows summarized information about the groups of 182 drugs which were administered to our study population. We categorized the drugs prescribed by the doctors of RCMPMC into 10 groups and examined. Antibiotics, vitamins-microelements and CV drugs were prescribed to the majority of patients (62.0, 80.0 and 64.0 % of all cases) with irrational usage in 88.0, 23.0 and 88.0 % of prescriptions. NSAIDs, antihistamines and glycosides were prescribed to around 40.0 % of children. The irrational usages of these were 85.0, 32.0 and 100 %, respectively. The following drugs were -prescribed in all cases irrationally and the percentage of their prescriptions were as follows: aminoglycosides (7.0 %), sulfonamides (0.8 %), fluoroquinolones (0.1 %), hydrochlorthiazide (0.1 %), hydrochlorthiazide + triamteren (1.0 %), β-adrenergic blocking agents (4.0 %), bendazol (0.1 %), piracetam + cinnarizin (0.5 %) and verapamil (0.1 %). Other most common medications prescribed irrationally were diclofenac (prescribed in 99.0 % cases, over-dose in 17.0 % of patients), prednisolone (96.0 %, over-dose in 19.0 % patients) and macrolides (96.0 %, over-dose in 12.0 % prescriptions). Amoxicillin, digoxin and potassium orotas were irrationally-prescribed in all cases being more than the required amount per kg. Only aspartic acid was prescribed within normal dose range in all cases.

**Table 3:** Prescribed drugs and irrational usage in patients with congenital and acquired heart diseases (*n* = 853)

Classification of drugs	Number of patients received drugs (%)	Number of patients received irrational drugs (%)^a^
NSAIDs^b^	349 (40.9)	298 (85.4)
Diclofenac	143 (16.8)	142 (99.3)
SAIDs^c^	201 (23.6)	192 (95.5)
Prednisolone	159 (18.6)	153 (96.2)
Vitamins-microelements	686 (80.4)	157 (22.9)
Ascorbic acid	215 (25.2)	10 (4.7)
Aspartic acid	461 (54.0)	0
Antibiotics	532 (62.4)	470 (88.3)
Cephalosporins	293 (34.3)	220 (75.1)
Penicillins	234 (27.4)	216 (92.3)
Amoxicillin	134 (15.7)	134 (100)
Aminoglycosides	65 (7.6)	65 (100)
Macrolides	98 (11.5)	94 (95.9)
Sulfonamides	7 (0.8)	7 (100)
Lincosamides	3 (0.4)	2 (66.7)
Fluoroquinolones	1 (0.1)	1 (100)
Antihistamines	379 (44.4)	122 (32.2)
Broncholitics	42 (4.9)	33 (78.6)
Diuretics	310 (36.3)	134 (43.2)
Furosemide	288 (33.8)	115 (39.9)
Hydrochlorothiazide	1 (0.1)	1 (100)
Hydrochlorothiazide + triamteren	8 (0.9)	8 (100)
Antispasmodics	32 (3.8)	22 (68.8)
Cardiovascular drugs	542 (63.5)	477 (88.0)
Glycosides	374 (43.8)	374 (100)
Digoxin	373 (43.7)	373 (100)
ACE inhibitors^d^	98 (11.5)	58 (59.2)
Captopril	91 (10.7)	58 (63.7)
β-adrenergic blocking agents	37 (4.3)	37 (100)
Propranolol	37 (4.3)	37 (100)
Other cardiovascular drugs	139 (16.3)	71 (51.1)
Bendazol	1 (0.1)	1 (100)
Piracetam + cinnarizin	4 (0.5)	4 (100)
Verapamil	1 (0.1)	1 (100)
Other drugs	327 (38.3)	221 (67.6)
Potassium orotas	115 (13.5)	115 (100)

^a^Of those who received the drug

^b^Non-steroid anti-inflammatory drugs

^c^Steroid anti-inflammatory drugs

^d^Angiotensin-converting enzyme inhibitors

All diuretics were prescribed for 36.0 % of patients (Table 3). Hydrochlorothiazide and hydrochlorothiazide + triamteren were administered in extra doses in 100 % of cases. CV drugs group included ACE inhibitors (captopril), β-adrenergic blocking agents (propranolol), calcium channel blocking agents (verapamil) and peripheral vasodilators (bendazol and piracetam + cinnarizine). ACE inhibitors were prescribed in 12.0 % of patients, and captopril was ordered irrationally in 64.0 % of all cases. Propranolol and verapamil were prescribed in 4.0 and 0.1 % of cases. Both drugs were used 100 % in extra doses. Peripheral vasodilators were used in 0.1 and 0.5 % of all cases. Piracetam + cinnarizine was used 100 % irrationally in 0.5 % administered cases.

**Fig. 1:**
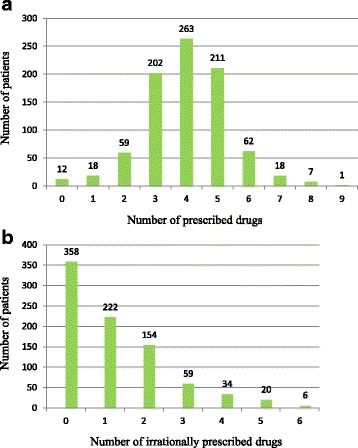
Frequency of patients receiving drugs (*n* = 853). (**a**) This figure illustrates frequency distribution of patients prescribed the drugs (*n* = 853); (**b**) This figure illustrates the number of patients prescribed rational and irrational drugs (*n* = 853)

Figure 1a shows frequency distribution of patients receiving drugs and Figure 1b illustrates the number of patients who were prescribed the drugs in irrationally high dose. Out of 853 patients, 358 (42.0 %) were not prescribed irrational drugs (overdose drugs). One drug over-usage was the most common prescription error (*n* = 222, 26.0 %) and less than 1 % of patients (*n* = 6) received the maximum 6 irrational medications. In our study 6 patients were prescribed 6 drugs with 4–6 in extra doses, 20 patients 5 drugs with 2–5 over-dosage and 34 patients 4 drugs with 3–4 extra-dosage.

Number of patients prescribed drugs and percentages of irrational prescriptions according to clinical and demographic characteristics are shown in Table 4. In the age group ≤1 years old, SAIDs, antibiotics, diuretics, antispasmodics and CV drugs were prescribed significantly more often. Their irrational usage was also higher at the age ≤1 years old in comparison to other age groups. Though antihistamines were not prescribed in more cases in this age group, their irrational usage was higher (29 %) than in older patients. NSAIDs, vitamins-microelements and diuretics were prescribed in fewer cases over-dose at the age ≤1 years old compared to other drugs and age groups. The length of hospitalization was also significantly associated with irrational prescription: the more days the patients spent in hospital, the higher is the possibility of over-dose prescription (especially for the drugs groups of vitamin-microelements, antispasmodics and other drugs). Patients whose weight is ≤10 kg also received more drugs irrationally in comparison to patients in other weight groups. Another not less significant factor for irrational prescription is the number of the prescribed drugs. In case of 7–14 drugs per prescription the irrational usage was significantly high for SAIDs, vitamins-microelements, antibiotics, bronchodilators, antispasmodics, CV drugs and the drugs in the group named “other drugs”. .

**Table 4:** Number of patients prescribed drugs and percentages of irrational prescriptions according to clinical and demographic characteristics (*n* = 853)

Characteristics	NSAIDs^a^	SAIDs^b^	Vitamins-microelements	Anti-biotics	Anti-histamines	Broncho-dilators	Diuretics	Anti-spasmodics	Cardiovascular drugs	Other drugs
Gender										
Male	184 (33)	111 (23)	371 (19)	303 (58)	202 (15)	21 (4)	168 (17)	18 (3)	299 (55)	175 (24)
Female	165 (37)	90 (23)	315 (18)	229 (52)	177 (13)	21 (4)	142 (15)	14 (2)	243 (57)	152 (28)
Residence										
Urban	70 (39)	32 (19)	122 (14)	102 (55)	86 (14)	10 (4)	42 (10)	5 (1)	84 (49)	62 (27)
Rural	279 (34)	169 (23)	564 (19)	430 (55)	293 (15)	32 (4)	268 (17)	27 (3)	458 (58)	265 (26)
Age (years)										
≤ 1	54 (**18**)	93 (**50**)	137 (**7**)	162 (**78**)	71 (**29**)	15 (**7**)	83 (9)	17 (**6**)	140 (**76**)	65 (25)
2–3	64 (**32**)	48 (**28**)	140 (**19**)	123 (**68**)	64 (**12**)	15 (**7**)	78 (16)	4 (**2**)	130 (**71**)	57 (21)
4–6	85 (**38**)	16 (**7**)	167 (**21**)	115 (**51**)	101 (**19**)	8 (**4**)	76 (16)	1 (**1**)	129 (**56**)	61 (22)
7–11	80 (**42**)	30 (**17**)	141 (**23**)	87 (**44**)	81 (**5**)	4 (**2**)	50 (22)	3 (**2**)	92 (**41**)	80 (32)
12–15	66 (**49**)	14 (**10**)	101 (**22**)	45 (**30**)	62 (**3**)	0	23 (16)	7 (**2**)	51 (**31**)	64 (30)
Length of hospitalization (days)										
1–7	150 (33)	75 (18)	326 (**17**)	253 (54)	174 (15)	15 (3)	153 (14)	8 (**1**)	266 (59)	136 (**22**)
≥ 8	199 (37)	126 (27)	360 (**20**)	279 (56)	205 (14)	27 (5)	157 (17)	24 (**4**)	276 (54)	191 (**30**)
Diagnosis										
Congenital heart diseases										
Surgical + medical	29 (**11**)	23 (**14**)	121 (24)	95 (58)	58 (15)	7 (4)	68 (19)	7 (3)	102 (54)	72 (34)
Medical	164 (**26**)	121 (23)	432 (20)	333 (59)	199 (20)	30 (5)	233 (**20**)	14 (2)	427 (**78**)	186 (26)
Acquired cardiovascular diseases										
Medical	191 (**25**)	158 (**26**)	520 (20)	394 (**59**)	218 (**16**)	34 (4)	294 (**22**)	27 (3)	503 (**77**)	243 (29)
Rheumatic diseases										
Medical	126 (**64**)	39 (19)	125 (15)	90 (**42**)	112 (**7**)	3 (**1**)	23 (**5**)	5 (2)	48 (**22**)	76 (27)
Weight (kg)										
≤ 10	72 (**18**)	117 (**46**)	191 (**10**)	215 (**76**)	95 (**28**)	21 (**6**)	111 (**9**)	17 (5)	196 (**77**)	83 (23)
11–19	130 (**39**)	45 (**15**)	249 (**21**)	179 (**57**)	134 (**14**)	18 (**6**)	126 (**15**)	4 (1)	202 (**60**)	101 (22)
≥ 20	147 (**44**)	39 (**12**)	246 (**23**)	138 (**38**)	150 (**4**)	3 (**1**)	73 (**21**)	11 (2)	144 (**37**)	143 (32)
Number of prescribed drugs										
1–4	124 (39)	32 (**10**)	189 (**11**)	139 (**39**)	119 (**8**)	7 (**2**)	41 (**7**)	3 (**1**)	112 (**31**)	77 (**13**)
5	85 (36)	29 (**14**)	185 (**22**)	115 (**46**)	82 (**14**)	7 (**3**)	86 (**19**)	2 (**1**)	153 (**66**)	75 (**25**)
6	62 (29)	47 (**26**)	158 (**21**)	133 (**68**)	95 (**21**)	8 (**3**)	88 (**24**)	6 (**3**)	146 (**75**)	71 (**31**)
7–14	78 (33)	93 (**53**)	154 (**26**)	145 (**81**)	83 (**20**)	20 (**10**)	95 (**18**)	21 (**8**)	131 (**69**)	104 (**45**)

Each value in parenthesis represents the percentage of patients with irrational drug prescriptions in each subgroup. Values in bold type indicate a significant difference (Chi-square test, *P* < 0.01) among the proportions of patients in the category receiving an irrational drug

^a^Non-steroid anti-inflammatory drugs

^b^Steroid anti-inflammatory drugs

## Discussion

Though there were many reviews of drug use by national and international authorized organizations country-specific guidelines for drug use still lack labeling, especially drugs for children with congenital and acquired heart diseases. Further more detailed and large scale researches are necessary to minimize the gap between the availability of drugs and real drug usage in children. Children are sometimes considered to be “small adults” [22] or “therapeutic orphans” [23], especially when some drugs are used for children’s treatment although they lack data for pediatric efficacy and safety [24].

Fifty eight percent of patients were prescribed over-dose drugs (irrational) in this study. Our results are not consistent with a study conducted in other countries in the Middle East which reported an incidence rate of prescribing errors from 0.15 to 34.8 %. The reason for irrational prescription may be the tendency of the doctors of RCMPMC in Andijan to poly-pharmacy that in their opinion may treat both main diseases and co-morbidities [4, 5, 7]. Yet, the difference in findings may be due to different methodology, scale of researches, and sample size [25-27]. With the increasing complexity of pharmacologic interventions with poly-pharmacy [28], the risk of medication errors may also increase [26]. The percentage of prescribed drugs depends on diagnosed conditions that may differ between countries or even regions [2]. In one study patients taking 10 or more drugs had nearly 50 % risk of error due to additive effect of each prescribed medicine at the same time [25]. Our study found that diclofenac, prednisolone, amoxicillin, digoxin, furosemide, captopril and potassium orotas were administered irrationally in 99.3, 96.2, 100, 100, 39.9, 63.7 and 100 % of cases, respectively. Doctors’ behavior in prescribing drugs contraindicated for pediatric use or prescribing more than indicated could be attributed to their lack of sufficient knowledge [1] for the use of selected drugs or may be due to their own practice based perception from observing the positive effects [22] of such “adult drugs” in children.

Diuretics formed one third of all antihypertensive prescriptions in our study whereas a previous study [21] found that the combination of diuretics and CV agents was 10 and 90 %, respectively. Doctors of RCMPMC in Andijan may have used diuretics and CV agents for antihypertensive effect. Hydrochlorthiazide and hydrochlorthiazide + triamteren (diuretics), propranolol (β-adrenergic blockers), verapamil (calcium channel blockers) and piracetam + cinnarizine (peripheral vasodilators) were administered in 100 % of cases irrationally because there are no indications for their administration at children’s age. These drugs might be considered the most appropriate ones by the doctors of RCMPMC in Andijan in respect of the severity of the disease.

Our analysis showed that several drugs pertaining to NSAIDs and antibiotics were unnecessarily combined in the treatment. However, SAIDs were not misused so much. Glycosides and antispasmodics were prescribed only by 1 type per person, whereas other CV drugs like β-adrenergic blocking agents, calcium channel blockers and peripheral vasodilators were more likely to be prescribed all together per one patient. Our literature review did not find relevant result in other studies conducted previously [7, 26]. In our study, significant amount of irrational prescriptions were among antibiotics, glycosides, NSAIDs, SAIDs and β-adrenergic blocking agents. The results of our study were consistent with those of the previous ones [2, 26]. The patients younger than 2-3 years old had the highest number of prescriptions in all groups of drugs (Table 4). The most vulnerable group were children aged 1–2 years and younger in previous studies [7, 26], which is consistent with our finding.

Treatment of congenital and acquired heart diseases by surgery or interventional procedures is always expensive and this may be one of main barriers on the way to achieving good cardiac care outcome [26]. Therefore, drug therapy may be an effective way to reach this aim, but will require improvement of treating schemes with minimal errors and maximal benefit for health. Novel approaches to building evidence-based treatment schemes, which generate sufficient information in real-time through retrospective analyses experience, may turn out to be a quite vital complement to clinical trials and classic cohort studies.

The following steps are suggested to bring improvement in evidence-based treatment practice in Uzbekistan. First, the interventional role of pharmacologists should be increased in decision making process for treatment of patients in each department by appointing responsible person from pharmacological background for pharmaceutical affairs management. Second, retrospective analyses should be conducted regularly (at least once per 2 years) to identify the current errors and their reasons and to evaluate the degree of improvement in response to suggested interventions. Third, medical personnel should be re-educated and it is necessary to make sure that they have up-to-date knowledge in line with all new national and international standard guidelines, dissemination of computerized information, and continuing pharmacological education.

Our study has some limitations. In this study we considered irrational prescription only from view point of weight and age. However, route of administration, performance of injections, and drug intake by patients that are considered to be other types of medication errors were not included in the study. We studied the PE of CV medications in only one department within one RCMPMC out of all existing medical centers in Uzbekistan. Although the results of this study may not be generalized to the situation in the whole country, they can highlight areas for further investigations of the same problem in all hospitals.

## Conclusion

In conclusion, The study found that irrational drug schemes were quite frequent among pediatric CV patients and they are most frequent in children aged 2–3 years and younger.

## Abbreviations

ACE: Angiotensin-converting enzyme
CHD: Congenital heart disease
CI: Confidence interval
CT: Clinical trial
CV: Cardiovascular
DUR: Drug utilization study or research
NSAIDs: Non-steroid anti-inflammatory drugs
PE: Pharmaco-epidemiology
RCMPMC: Regional Children’s Multi-Profile Medical Centre
SAIDs: Steroid anti-inflammatory drugs
SPSS: Statistical package for social science
